# Association of hospital volume with perioperative and oncological outcomes of robot-assisted laparoscopic radical prostatectomy: a retrospective multicenter cohort study

**DOI:** 10.1186/s12894-023-01178-w

**Published:** 2023-01-31

**Authors:** Tomoyuki Tatenuma, Shin Ebara, Makoto Kawase, Takeshi Sasaki, Yoshinori Ikehata, Akinori Nakayama, Masahiro Toide, Tatsuaki Yoneda, Kazushige Sakaguchi, Jun Teishima, Takahiro Inoue, Hiroshi Kitamura, Kazutaka Saito, Fumitaka Koga, Shinji Urakami, Takuya Koie, Kazuhide Makiyama

**Affiliations:** 1 Department of Urology, Yokohama City University Hospital, 3-9 Fukuura, Kanazawaku, Yokohama, Kanagawa 2360004 Japan; 2 Department of Urology, Hiroshima City Hiroshima Citizens Hospital, Hiroshima, Japan; 3 Department of Urology, Gifu University Graduate School of Medicine, Gifu, Japan; 4 Department of Nephro-Urologic Surgery and Andrology, Mie University Graduate School of Medicine, Tsu, Japan; 5 Department of Urology, University of Toyama, Toyama, Japan; 6 Department of Urology, Dokkyo Medical University Saitama Medical Center, Koshigaya, Japan; 7 Department of Urology, Tokyo Metropolitan Cancer and Infectious Diseases Center Komagome Hospital, Tokyo, Japan; 8 Department of Urology, Seirei Hamamatsu General Hospital, Hamamatsu, Japan; 9 Department of Urology, Toranomon Hospital, Tokyo, Japan; 10 Department of Urology, Graduate School of Biomedical and Health Science, Hiroshima University, Hiroshima, Japan

**Keywords:** Prostate cancer, Robot-assisted radical prostatectomy, High-volume hospital, Retrospective multicenter cohort study

## Abstract

**Background:**

This retrospective multicenter cohort study investigated the association of hospital volume with perioperative and oncological outcomes in patients treated with robot-assisted radical prostatectomy (RARP).

**Methods:**

We collected the clinical data of patients who underwent RARP at eight institutions in Japan between September 2012 and August 2021. The patients were divided into two groups based on the treatment site—high- and non-high-volume hospitals. We defined a high-volume hospital as one where RARP was performed for more than 100 cases per year.

**Results:**

After excluding patients who received neoadjuvant therapy, a total of 2753 patients were included in this study. In the high-volume hospital group, console time and estimated blood loss were significantly (p < 0.001) lower than that of the non-high-volume hospital group. However, the continence rate at 3 months after RARP, positive surgical margins, and prostate-specific antigen (PSA)-relapse-free survival showed no significant differences between the two groups. Furthermore, the console time was significantly shorter after 100 cases in the non-high-volume hospital group but not in the high-volume hospital group.

**Conclusions:**

A higher hospital volume was significantly associated with shorter console time and less estimated blood loss. However, oncological outcomes and early continence recovery appear to be comparable regardless of the hospital volume in Japan.

**Supplementary Information:**

The online version contains supplementary material available at 10.1186/s12894-023-01178-w.

## Background

Radical prostatectomy is one of the treatment options for localized prostate cancer; robot-assisted radical prostatectomy (RARP) has been widely used in Japan because it is associated with better perioperative outcomes than laparoscopic radical prostatectomy (LRP) and radical retropubic prostatectomy (RRP) [1, 2]. In Japan, there are only a few high-volume centers in which more than 100 cases per year of RARP are performed. Although perioperative outcomes of open radical prostatectomy in high-volume centers and the association between the hospital volume and outcomes have been reported [3, 4], the relationships among hospital volume, biochemical recurrence, and urinary continence are poorly understood. In this study, we investigated the association of hospital volume with perioperative and oncological outcomes in patients treated with RARP.

## Methods

### Patients

In this retrospective, multicenter cohort study, patients with prostate cancer who underwent RARP at eight institutions in Japan between September 2012 and August 2021 were enrolled. The patients were divided into high- (> 100 RARPs per year) and non-high-volume hospital groups. Patient-related information was collected including as age, body mass index (BMI), initial prostate-specific antigen (PSA) level, clinical T stage, D'Amico classification risk, console time, blood loss, pathological stage, Gleason score, nerve-sparing, and pelvic lymph node dissection (PLND). The presence or absence of PLND, range of PLND, and nerve-sparing approach were determined according to the policies of each institution. Serum PSA levels of > 0.2 ng/mL were defined as the date of PSA failure; when the PSA levels did not drop below 0.2 ng/mL; the date of RARP was defined as the date of disease recurrence or PSA persistence. Further, urinary continence was limited to the use of a single safety pad. We performed perioperative treatment and follow-up based on a protocol that was standardized across institutions.

### Statistical analysis

The univariate analysis was used to compare the high- and non-high-volume hospital groups. Unpaired t-tests and the chi-squared test were used to compare continuous and categorical variables, respectively. Multiple linear regression analysis was used for the multivariate analysis to identify factors associated with the perioperative outcomes. Biochemical recurrence-free survival after RARP was analyzed using the Kaplan–Meier method. Cox regression analysis was used for the multivariate analysis to identify factors associated with biochemical recurrence. Statistical significance was set at p < 0.05. All the statistical analyses were conducted using SPSS version 20.

## Results

### Patient characteristics

Of the 3195 enrolled patients, those who received neoadjuvant therapy were excluded and 2753 patients were finally included in this study. Table 1 shows the number of RARPs performed per year at each hospital; among the total hospitals analyzed, three were classified as high-volume hospitals. Table 2 shows the patients’ characteristics of both groups. Age, initial PSA level, clinical T stage, and prostate volume differed significantly between the two groups.

**Table 1:** The proportion of robot-assisted radical prostatectomies (RARPs) performed per year at each hospital (A) and the surgeon’s experience of RARP in each group (B)

	Cases/year	Number of surgeons	
*(A)*			
High-volume hospitals			
Hospital A	102.2	10	
Hospital B	110.0	8	
Hospital C	117.0	13	
Non-high-volume hospitals			
Hospital D	40.9	4	
Hospital E	45.8	4	
Hospital F	61.5	6	
Hospital G	66.1	10	
Hospital H	69.0	3	

	High-volume hospitals	Non-high-volume hospitals	P value
*(B)*			
< 40 cases	16 (51.6%)	11 (42.3%)	0.59
40–99	8 (25.8%)	10 (38.5%)	
≥ 100	7 (22.6%)	5 (19.2%)

**Table 2:** Patients’ characteristics

	High-volume hospital (n = 1842)	Non-high-volume hospital (n = 911)	P value
Age, years (median ± SD)	69 ± 6.0	68 ± 6.1	0.001
BMI (median ± SD)	23.6 ± 2.9	23.6 ± 3.0	0.68
Initial PSA ng/mL (median ± SD)	7.9 ± 7.8	7.0 ± 6.0	< 0.001
Clinical T stage (number, %)			
T1	406 (22.0%)	137 (15.0%)	< 0.001
T2	1303 (70.7%)	696 (76.4%)	
T3	131 (7.1%)	78 (8.6%)	
Biopsy Gleason score (number, %)			
6	403 (21.9%)	188 (20.6%)	0.80
7	958 (52.0%)	481 (52.8%)	
8–10	480 (26.1%)	242 (26.6%)	
D’Amico risk classification (number, %)			
Low	249 (13.5%)	101 (11.1%)	0.054
Intermediate	824 (44.7%)	391 (42.9%)	
High	769 (41.7%)	419 (46.0%)	
Prostate volume, mL (median ± SD)	30.2 ± 15.7	28.0 ± 17.2	0.02
Observation period, months (median ± SD)	30.4 ± 26.7	17.6 ± 16.5	< 0.001

### Surgical outcomes

**Fig. 1:**
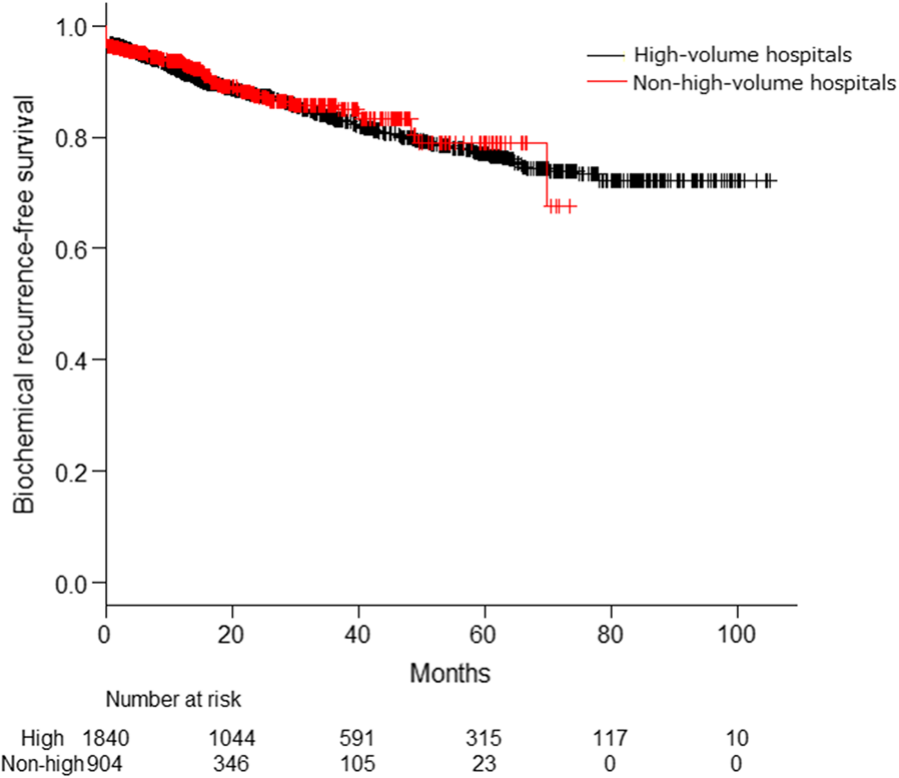
Kaplan–Meier curve for estimated biochemical recurrence-free survival according to hospital volume. No significant differences between high- and non-high-volume hospitals were found

Table 3 shows the surgical outcomes of both groups. The console time and estimated blood loss in the patients of the high-volume hospital group were significantly less than that of the patients in the non-high-volume hospital group (p < 0.001). Patients in the high-volume hospital group underwent less expanded lymph node dissection (with a smaller number of lymph nodes) relative to those in the non-high-volume hospital group. PSA persistence rate in the high-volume hospital group was lower than that in the non-high-volume hospital group. However, no significant differences in biochemical recurrence-free survival were found (Fig. 1). No significant difference was noticed between the two groups in terms of the continence rate at 3 months after RARP, complication rates, and rate of positive surgical margins (Table 3). More details on these complications are shown in the Additional file 1: Table S1. On multivariable analysis, preoperative PSA values, pathological Gleason score, pathological T stage, pathological lymph nodal status, and surgical margin status were independently associated with biochemical recurrence (Table 4). However, the hospital volume did not show a prognostic significance.

**Table 3:** Surgical and pathological outcomes

	High volume hospital (n = 1842)	Non-high-volume hospital (n = 911)	P value
Console time, min (median ± SD)	146 ± 57	203 ± 74	< 0.001
Estimated blood loss, mL (median ± SD)	30 ± 182	169 ± 271	< 0.001
Nerve spare (number, %)			< 0.001
Unilateral	396 (21.5%)	240 (26.3%)	
Bilateral	53 (2.9%)	138 (15.1%)	
Not performed	1391 (75.5%)	533 (58.5%)	
Lymph node dissection			< 0.001
Limited dissection	1085 (58.9%)	464 (50.9%)	
Extended dissection	169 (9.2%)	153 (16.8%)	
Not performed	584 (31.7%)	288 (31.6%)	
Number of lymph nodes (extended dissection)	14 ± 7.8	18 ± 8.2	< 0.001
Pathological T stage (number, %)			0.11
T2	1273 (69.1%)	656 (72.0%)	
T3 or more	566 (30.7%)	253 (27.8%)	
Pathological nodal status (number, %)			0.28
N0	1187 (94.6%)	591 (95.6%)	
N1	67 (5.3%)	26 (4.2%)	
Surgical margins status (number, %)			0.29
Negative	1259 (68.3%)	615 (67.5%)	
Positive	560 (30.4%)	249 (27.3%)	
Surgical Gleason score (number, %)			0.16
6	131 (7.1%)	74 (8.1%)	
7	1339 (72.7%)	627 (68.8%)	
8–10	368 (20.0%)	203 (22.3%)	
Complication (number, %)			0.9
Grade 3	46 (2.5%)	21 (2.3%)	
Grade 4	3 (0.2%)	2 (0.2%)	
PSA persistence	77 (4.2%)	59 (6.5%)	0.003
Continence rate at 3 months after RARP	686 (37.4%)	189 (37.0%)	0.93

**Table 4:** Multivariate regression analysis associated with biochemical recurrence

	Hazard ratio (95% CI)	P value
Preoperative PSA (≥ 7.4 ng/ml)	1.55 (1.22–1.98)	< 0.001
Nerve sparing (performed)	0.81 (0.62–1.05)	0.11
Lymph node dissection (performed)	1.22 (0.45–3.31)	0.68
Pathological Gleason score (8–10)	2.55 (2.02–3.21)	< 0.001
Pathological T stage (pT3 or T4)	1.92 (1.48–2.48)	< 0.001
Pathological lymph nodal status (positive)	3.81 (2.81–5.16)	< 0.001
Surgical margin status (positive)	1.93 (1.52–2.46)	< 0.001
Hospital volume (high-volume)	1.00 (0.77–1.31)	0.95

Multivariate analysis showed that the hospital volume, BMI, and prostate volume were independent significant (p < 0.001) factors associated with both the console time and estimated blood loss (Table 5). Further, in the high-volume hospital group, no significant difference in console time was noticed before and after 100 cases. However, the console time was significantly shorter after 100 cases in the non-high-volume hospital group compared to the first 100 cases (Table 6).

**Table 5:** Multiple linear regression analysis examining the console time (A) and the estimated blood loss (B)

	Beta (95% CI)	P value
(A)		
Age	− 0.70 (− 1.09 to − 0.31)	< 0.001
BMI	2.28 (1.48–3.09)	< 0.001
Risk classification	11.3 (7.71–15.0)	< 0.001
Nerve spare	3.27 (− 0.82 to 7.39)	0.11
Prostate volume	0.65 (0.50–0.80)	< 0.001
Lymph node dissection	4.54 (− 0.92 to 10.02)	0.10
Hospital volume	61.4 (56.2–66.6)	< 0.001
(B)		
Age	− 1.65 (− 2.99 to − 0.31)	0.015
BMI	13.9 (11.1–16.6)	< 0.001
Risk classification	5.09 (− 7.47 to 17.6)	0.42
Nerve spare	39.6 (25.5–53.6)	< 0.001
Prostate volume	2.28 (1.78–2.78)	< 0.001
Lymph node dissection	12.2 (− 6.42 to 30.9)	0.19
Hospital volume	167 (149–185)	< 0.001

**Table 6:** Comparison of console time and estimated blood loss in the first 100 cases and beyond the first100 cases

	First 100 cases	After 100 cases	P value
High-volume hospitals			
Console time, min (median ± SD)	142 ± 72	146 ± 54	0.10
Estimated blood loss, mL (median ± SD)	30 ± 165	30 ± 185	0.86
Non-high-volume hospitals			
Console time, min (median ± SD)	225 ± 82	192 ± 64	< 0.001
Estimated blood loss, mL (median ± SD)	150 ± 272	200 ± 269	0.23

## Discussion

In this study, through the analysis of long-term data including the biochemical recurrence failure in patients who underwent RARP, we analyzed the clinical outcomes of patients treated at high- and non-high-volume hospitals. Although the non-high-volume hospitals showed a longer console time and more blood loss, the oncological and short-term incontinence outcomes were comparable to that of the high-volume hospitals.

PSA persistence rate in high-volume hospitals was lower than that in non-high-volume hospitals. Bianchi et al. [5] showed that PSA persistence (PSA ≥ 0.1 ng/ml) 6 weeks after radical prostatectomy and PLND were independent predictors of both clinical recurrence and cancer-specific mortality in patients with lymph node invasion. Although we did not find a correlation between PSA persistence and long-term prognosis, PSA persistence may be related to surgical quality.

Previously, only a few studies have analyzed the relationship between hospital volume and RARP outcomes. Budaus et al. [6] reported the relationship between surgeon volume and minimally invasive surgery, including RARP, for the first time. Hirasawa et al. [7] showed that hospital volume is a significant risk factor for perioperative complications. Further, in a study by Xia et al. [8] a higher hospital volume was associated with lower odds of conversion to open surgery, prolonged length of stay, 30-day readmission, and positive surgical margins. All reports suggest that high-volume hospitals have better perioperative outcomes relative to low-volume hospitals. However, these reports have often been analyzed using a national database. Therefore, the observation period was relatively short, and there was little information about recurrence and postoperative complications, such as urinary incontinence. There were some reports about the relationships between hospital volume, oncological outcomes, and urinary complications for open radical prostatectomy. Ellison et al. [9] reported an increased risk of adjuvant therapy with medium- and low- versus high-volume hospitals about RRP. Furthermore, Begg et al. [10] reported that an increased hospital volume was related to reduced rates of postoperative and late urinary complications but not to reduced rates of long-term incontinence about RRP. However, the relationships among hospital volume, biochemical recurrence, and urinary continence of RARP are poorly understood. Therefore, the current study is a valuable report showing these outcomes.

Conventionally, RARP is considered to have a short learning curve. Patel et al. [11] reported a learning curve of approximately 20–25 cases. However, we found that the learning curve is individual-dependent, and the RARP requires more experience to achieve desirable results in practice. Slusarenco et al. [12] reported that the median operative time decreased after the 88th case. Similarly, Doumerc et al. [13] reported that 110 cases would be required to achieve an operative time of 180 min. We investigated the console time in the first 100 and after 100 cases in both the study groups, and found no significant difference in the high-volume hospitals. Hence, the console time in high-volume hospitals is stable from the early stages of introduction, suggesting that the learning curve of high-volume hospitals may be shorter than that of non-high-volume hospitals. Wang et al. [14] reported that the perioperative outcomes, after adding a newly trained surgeon to a high-volume hospital, were not compromised by the learning curve. We investigated the number of surgeons per hospital and the surgeon’s experience with RARP as shown in Table 1. This analysis suggested that non-high-volume hospitals also had experienced surgeons, comparable to those in high-volume hospitals. However, the number of cases that each surgeon performed and experienced with LRP was not included in this study. We must consider the presence of an expert in a high-volume hospital. For surgeons trained in 200–300 LRP procedures, the median operative time for RARP rapidly reduced, and the learning curve was 20 cases [15]. Therefore, further studies are required with consideration of the surgeon’s volume and experience in such analysis.

This study had some limitations. First, the data were collected from only eight institutions and may have caused a selection bias. Therefore, further studies with data from a large number of institutions need to be conducted. Second, this study alone could not determine whether the RARP should be centralized. For example, the initiation of a quality assurance program in London could improve urinary continence 3 months post-surgery [16]. In Japan, Hirasawa et al. [7] reported that surgeon volume was a significant risk factor for perioperative complications. Here, the authors conducted the study using data of RARP performed between 2012 and 2013. Subsequently, the RARP has been frequently implemented in Japan. Additionally, according to the guidelines to start RARP in Japan, the first few operations at each hospital must be proctored by certified experienced surgeons. The proctoring system in Japan may be attributed, in part, to comparable outcomes relevant to the quality measurement of RARP (e.g., oncological outcomes, early continence recovery, and complication rates) between high-volume and non-high-volume hospitals in our study.

## Conclusions

The present study demonstrated that a higher hospital volume was significantly associated with shorter console time and less estimated blood loss, suggesting an association between the hospital volume and learning curves. However, surgical quality appears to be comparable between the high- and non-high-volume hospitals in terms of surgical complications, oncological outcomes, and early continence recovery.

## Supplementary Information

**Additional file 1.**

## Data Availability

The datasets used and analyzed during the current study are available from the corresponding author upon reasonable request.

## Abbreviations

BMI: Body mass index
LRP: Laparoscopic radical prostatectomy
PLND: Pelvic lymph node dissection
PSA: Prostate-specific antigen
RARP: Robot-assisted radical prostatectomy
RRP: Radical retropubic prostatectomy
