# Supplementary material for: Association of hospital volume with perioperative and oncological outcomes of robot-assisted laparoscopic radical prostatectomy: a retrospective multicenter cohort study
Source: BMC Urol. 2023 Jan 31;23:14. doi: 10.1186/s12894-023-01178-w (PMC9887802; doi:10.1186/s12894-023-01178-w)
Supplement: Supplementary file 1 — Additional file 1. [file 12894_2023_1178_MOESM1_ESM.docx]

**Supplementary Table. Perioperative complications of grade3 or higher in each group**

|  | High volume hospital  (n=1842) | Non-high-volume hospital (n=911) |
| --- | --- | --- |
| Anastomotic leakage  Wound dehiscence  Hemorrhage  Intestinal injury  Lymphocele  Cardiovascular events  Urinary retention  Surgical site infection  Ileus  Abdominal incisional hernia  Rectal injury  Bladder injury  Compartment syndrome  Urethral stricture  Others | 8 (0.4%)  7 (0.3%)  6 (0.3%)  5 (0.2%)  4 (0.2%)  3 (0.1%)  3 (0.1%)  2 (0.1%)  2 (0.1%)  2 (0.1%)  1 (0.05%)  1 (0.05%)  1 (0.05%)  0  4 (0.2%) | 4 (0.4%)  0  2 (0.2%)  1 (0.1%)  6 (0.6%)  0  0  1 (0.1%)  3 (0.3%)  1 (0.1%)  1 (0.1%)  0  1 (0.1%)  2 (0.2%)  1 (0.1%) |
